# Cloud Model-Based Artificial Immune Network for Complex Optimization Problem

**DOI:** 10.1155/2017/5901258

**Published:** 2017-05-24

**Authors:** Mingan Wang, Shuo Feng, Jianming Li, Zhonghua Li, Yu Xue, Dongliang Guo

**Affiliations:** ^1^School of Information Science and Technology, Huizhou University, Huizhou 516007, China; ^2^School of Electronic Information and Electrical Engineering, Huizhou University, Huizhou 516007, China; ^3^School of Electronics and Information Technology, Sun Yat-sen University, Guangzhou 510006, China; ^4^School of Computer and Software, Nanjing University of Information Science & Technology, Nanjing 210044, China; ^5^Public Experimental Teaching Center, Sun Yat-sen University, Guangzhou 510006, China

## Abstract

This paper proposes an artificial immune network based on cloud model (AINet-CM) for complex function optimization problems. Three key immune operators—cloning, mutation, and suppression—are redesigned with the help of the cloud model. To be specific, an increasing half cloud-based cloning operator is used to adjust the dynamic clone multipliers of antibodies, an asymmetrical cloud-based mutation operator is used to control the adaptive evolution of antibodies, and a normal similarity cloud-based suppressor is used to keep the diversity of the antibody population. To quicken the searching convergence, a dynamic searching step length strategy is adopted. For comparative study, a series of numerical simulations are arranged between AINet-CM and the other three artificial immune systems, that is, opt-aiNet, IA-AIS, and AAIS-2S. Furthermore, two industrial applications—finite impulse response (FIR) filter design and proportional-integral-differential (PID) controller tuning—are investigated and the results demonstrate the potential searching capability and practical value of the proposed AINet-CM algorithm.

## 1. Introduction

Biological immune system (BIS), as one of the most complex body systems, plays a significant important role in protecting our bodies from the invasion of a large variety of external bacteria, viruses, and other pathogenic organisms. Inspired by BIS, artificial immune system (AIS) has been developed as an efficient optimization method, which has great computational potentials in solving scientific computing and engineering application problems [1, 2].

The variations of AIS mainly consist of four branches of theories/models: clone selection theory [3], negative selection theory [4], danger theory [5], and artificial immune network [6]. Specifically speaking, artificial immune network is widely used in a great number of applications, such as data analysis [7], function optimization [8], signal and image processing [9], process control [10], and Internet of Things [11]. De Castro and Timmis developed an earlier version of artificial immune network. Then, it was modified and called opt-aiNet [8], which is able to maintain stable local optima solutions in solving multimodal function optimization. Inspired by omni-optimization and immune evolution, omni-aiNet [12] was proposed with dynamical population size and low redundancy. By absorbing the elitist-learning strategy of particle swarm optimization (PSO), aiNet-EL [13] is able to discriminate the elitist antibodies and the other common antibodies during the mutation operation. By redesigning three major operators, the affinity-based cloning, the affinity-based mutation, and the concentration-based suppressor, IA-AIS [14] has more adaptability to field problems. Guided by elitist antibodies, AAIS-2S [15] divides the antibodies into two subpopulations: an elitist swarm with self-learning and a common swarm with elitist-learning. These achievements suggest that artificial immune network is becoming an active and hot research field.

Cloud model [16] is a conversion model with certainty between a qualitative concept and a quantitative number expression. Up to date, cloud model has been applied in many fields [17, 18] due to its randomness and stability. For example, in intelligent computation, several cloud model-based algorithms—the cloud-based adaptive genetic algorithm (CAGA) [19], asymmetrical cloud model-based genetic algorithm (ACGA) [20], and particle swarm optimization with normal cloud model (CPSO) [21]—have been developed. It is clearly shown that the combination of the cloud model and evolutionary algorithms is of interest to researchers and engineers.

The article [22] proposes an artificial immune network based on the cloud model (AINet-CM), where the cloud models are used to evaluate the candidate antibodies. Different cloud models are embedded into three major immune operators—clone, mutation, and suppression—to enhance the algorithmic convergence. As an extensive study of [22], this paper will systematically investigate the cloud-based operators of AINet-CM and examine the convergence and accuracy of AINet-CM by evaluating unimodal or multimodal functions whose dimension is 2D, 10D, and 30D. In addition, two kinds of typical applied experiments—band-pass FIR filter designing and industrial PID controller optimization—are arranged to demonstrate the effectiveness and high-performance of AINet-CM.

The remainder of this paper is organized as follows.  Section 2 reviews the principles of three artificial immune network family members and a cloud model.  Section 3 describes the technical details of the proposed AINet-CM algorithm.  Section 4 makes some comparisons in solution accuracy and convergence speed between the proposed AINet-CM algorithm and the other three artificial immune networks by a series of numerical simulations.  Section 5 describes and explains the experimental results obtained by four immune algorithms from FIR band-pass filter designing and PID controller parameter tuning. Finally, the conclusions are made in Section 6.

## 2. Reviews of Artificial Immune Network and Cloud Model

### 2.1. The Opt-aiNet Optimization

In opt-aiNet [8], all the antibodies experience five phases: clone, mutation, selection, suppression, and recruitment. At each generation, each individual parent antibody is cloned for a fixed number Nc and all the cloned offspring but the parent one must go through mutation operator. In this case, the mutated antibody with the highest fitness is selected to enter the next generation. If the average affinity among the current population is not significantly different from that among the previous population, the suppression process will be activated. If the Euclidean distance between any two antibodies is less than a threshold value Th_*s*_, the antibody with lower affinity will be suppressed or abandoned. And then, a certain percentage of randomly generated antibodies are recruited. The iterative process is repeated until the stopping criterion is met. Many researches demonstrated that the opt-aiNet is good at exploration but weak in exploitation for accurate solutions.

### 2.2. The IA-AIS Optimization

To improve the adaptability of parameters in opt-aiNet, an improved adaptive AIS (IA-AIS) algorithm [14] is proposed. In IA-AIS, the cloning operator depends on the affinity measure. In other words, the number of cloned antibodies is nonlinearly determined by the normalized affinity. And a controlled Gaussian mutation operator is able to make the mutation level decrease sharply as the affinity increases. Moreover, a concentration-based suppressor can adjust dynamically the suppression threshold, because this threshold is proportional to the similarity of antibodies. However, IA-AIS pays little attention to the improvement in solution accuracy during the iterative process.

### 2.3. The AAIS-2S Optimization

In PSO, it is well known that the particle always learns from the best particle. Inspired by this elitist-learning strategy of PSO, AAIS-2S [15] separates the population into two subgroups: an elitist swarm (ES) and a common swarm (CS), where ES is able to go through self-learning mutation while CS is required to learn from the best antibody in ES. Meanwhile, a swarm updating mechanism is added to make those better antibodies in CS upgrade into ES. In addition, the searching step length is adjusted dynamically according to the Euclidean distance measure between antibodies. As a result, AAIS-2S can obtain the global optima quickly but has a potential risk in getting trapped into the local optimum.

### 2.4. The Cloud Model

The cloud model is a transforming model between qualitative concepts and their quantitative expressions. Assume that *U* is defined as a quantitative universe expressed by numerical value and *C* is defined as a qualitative concept in *U*. When the quantitative value *x* ∈ *U* means a specific random realization of *C*, the certainty degree of *x* related to qualitative concept *C* can be expressed as *μ*(*x*)∈[0,1]. Then, *μ*(*x*)∈[0,1] is a random number with the stable tendency:

(1) $$\begin{array}{c} \mu :U⟶\left[\;0,1\;\right]\;\forall x\in U,\;\;x⟶\mu \left(x\right). \end{array}$$

**Figure 1:**
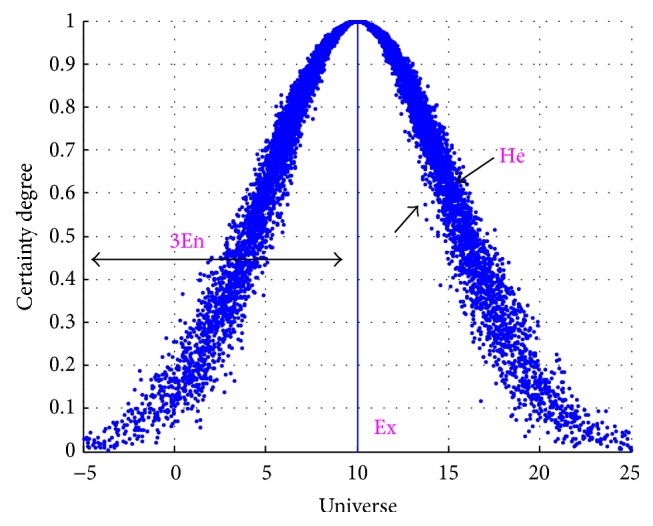
A normal cloud with (Ex, En, He) = (10,5, 0.5).

The distribution of *x* in the universe *U* is called* cloud* in term, and each (*x*, *μ*) is called a* cloud drop* [16]. A simple normal cloud is illustrated in Figure 1. Known from the definition above and Figure 1, it is obvious that the mapping from *U* to interval [0,1] is equivalent to the one-point to multipoint transition with certainty by integrating fuzzy degree and randomness.

For a specific cloud, its characteristics can be measured by three parameters, that is,* expectation* (Ex),* entropy* (En), and* hyperentropy* (He), where Ex denotes the expectation value of the distribution of the cloud drops in the universe and is the typical swatch among the cloud. En is a measure of the coverage of the qualitative concept within the universe, which determines the range of the cloud. He is the entropy of En, which is decided by both randomness and fuzzification. Therefore, a cloud model can be built by these three parameters (Ex, En, He). Figure 1 illustrates a normal cloud with the numerical characteristics value (10,5, 0.5).

## 3. The Proposed Artificial Immune Network Based on Cloud Model (AINet-CM)

With the help of the cloud model, it is possible that artificial immune network escapes from getting trapped into the local optimum by using the diversity and the stability of cloud model measure. Thus, it is advantageous for the candidate antibodies to evolve approximately to the global optimum. The technical details of the proposed artificial immune network based on cloud model (AINet-CM) optimization are described as follows.

### 3.1. Cloning Operator

Suppose that *N* antibodies with real number coding have been randomly generated in the initialization phase. Then, the *i*th antibody in the population at the *t*th generation can be defined as Ab(*i*, *t*)  (*i* = 1,…, *N*). Generally speaking, in this cloning phase, the greater the parent antibody's affinity is, the more the cloning offspring antibodies are. It is clear that the cloned multiplier is positively related to the parent antibody's affinity.

**Figure 2:**
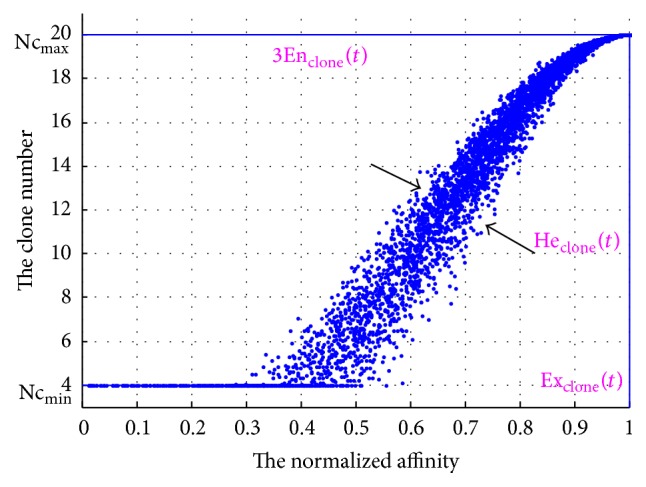
The increasing half cloud-based cloning operator (cloud chart) (see also Pseudocode 1).

To make sure that the antibodies with higher affinity should have larger clone multiplier, an increasing half cloud shown in Figure 2 is introduced. The characteristics of this cloud can be obtained by

(2) Exclonet=Affmax∗t,Enclonet=Affmax∗t−Affmin∗tcclone1,Heclonet=Enclonetcclone2,

where Aff_max_^*∗*^(*t*) and Aff_min_^*∗*^(*t*) represent the maximum and minimum normalized affinity in the interval [0,1] at the *t*th generation, respectively, and *c*_clone_^1^ and *c*_clone_^2^ are controlling parameters. While generating the increasing half cloud, Ex_clone_(*t*) should be equal to the maximum affinity value, which guarantees that the clone multiplier is larger for the antibodies with higher affinity. In addition, En_clone_(*t*)—which is controlled by parameter *c*_clone_^1^—decides the range of the increasing half cloud. If En_clone_(*t*) is too small, the cloud will look narrow and most clone multipliers will be limited to the lower boundary. On the contrary, if En_clone_(*t*) is too large, the cloud will be wide and most clone multipliers will be close to the upper boundary. Based on the “3En” rule [16], *c*_clone_^1^ = 3 is chosen in this paper. He_clone_(*t*)—another characteristic controlled by the parameter *c*_clone_^2^—decides the dispersion of the cloud. To be specific, too great He_clone_(*t*) will lose the stable tendency of cloud model to some extent, while too small He_clone_(*t*) will partly lose the randomness. According to the paper [20], the best range for *c*_clone_^2^ equals [6, 15]. So, we choose *c*_clone_^2^ = 10 to evaluate He_clone_(*t*) in this paper.

After the increasing half cloud is formed, the certainty degree of individual antibody can be obtained. Then, in the proposed AINet-CM algorithm, an* increasing half cloud-based cloning operator (IHC-based cloning operator) *

(3) $$\begin{array}{c} Nc\left(\;i,t\;\right)=max\left(\;Nc_{max}\cdot \mu \left(\;i,t\;\right),Nc_{min}\;\right) \end{array}$$

is used to determine the clone multiplier for each antibody individual. In (3), Nc_max_ and Nc_min_ are the upper and the lower bounds of the clone multiplier, respectively, and *μ*(*i*, *t*) represents the certainty degree of the *i*th antibody at the *t*th generation. For the sake of clarity, Pseudocode 1 shows pseudocode of IHC-based cloning operator and Figure 2 illustrates an increasing half cloud for Nc_max_ = 20 and Nc_min_ = 4.

### 3.2. Mutation Operator

**Figure 3:**
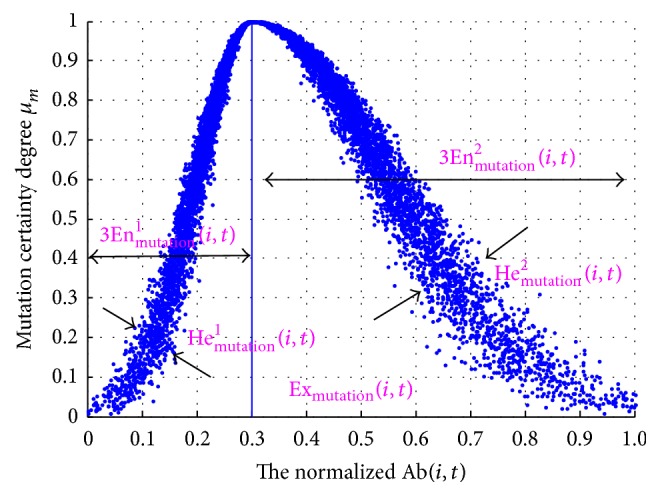
The asymmetrical cloud-based mutation operator (cloud chart) (see also Pseudocode 2).

To direct the mutation process, an* asymmetrical cloud-based mutation operator (AC-based mutation operator)* is well designed, which uses an asymmetrical cloud shown in Figure 3. The asymmetrical cloud consists of the left half and right half clouds, and their numerical characteristics can be marked as (Ex_mutation_(*i*, *t*), En_mutation_^1^(*i*, *t*), He_mutation_^1^(*i*, *t*)) and (Ex_mutation_(*i*, *t*), En_mutation_^2^(*i*, *t*), He_mutation_^2^(*i*, *t*)), respectively. The expectation Ex_mutation_(*i*, *t*) of the asymmetrical cloud denotes the mutation bit of the *i*th antibody at the *t*th generation. The entropy En_mutation_(*i*, *t*) represents the range of the searching step length for the *i*th antibody at the *t*th generation, and its left half and right half parts are marked as En_mutation_^1^(*i*, *t*) and En_mutation_^2^(*i*, *t*), respectively. Their expressions are given as

(4) Enmutation1i,t=13Exmutationi,t−Vmin·βi,t,Enmutation2i,t=13Vmax−Exmutationt·βi,t,

where *V*_min_ and *V*_max_ represent the lower bound and upper bounds of mutation scale, respectively. According to the “3En” rule, (4) guarantees that the mutated antibody Ab(*i*, *t* + 1) always lies within its range of domain. In addition, *β*(*i*, *t*) is the controlled coefficient of searching step length, which can be obtained by

(5) βi,t=βi,t−1,if  AffAbi,t>AffAbi,t−1,βi,t−1·K,elseif  βi,t−1·K>βmin,β0,T+1,otherwise,β0,T=β0,T−1·K,if  T>0,  β0,T·K≥βmin,β0,otherwise,

where *K* is a controlled parameter in (0,1), Aff(Ab(*i*, *t*)) represents the affinity of Ab(*i*, *t*), *β*_min_ denotes the minimum coefficient of the searching step length, and *β*(0) is the initialized value in (0,1). From (5), the searching step length will decrease if Ab(*i*, *t*) has no improvement in affinity after mutation. However, both *β*(*i*, *t*) and *β*(0, *T*) have to be greater than or equal to *β*_min_.

Moreover, He_mutation_^1^(*i*, *t*) and He_mutation_^2^(*i*, *t*)—the left half part and right half part of hyperentropy—are determined by

(6) Hemutation1i,t=Enmutation1i,tcmutation1,Hemutation2i,t=Enmutation2i,tcmutation2,

where *c*_mutation_^1^ and *c*_mutation_^2^ are the controlling parameters. To keep the stability and the randomness, *c*_mutation_^1^ and *c*_mutation_^2^ should not be too large or too small. According to the paper [20], the best range for *c*_mutation_^1^ and *c*_mutation_^2^ is [6, 15], so *c*_mutation_^1^ = *c*_mutation_^2^ = 10 is adopted in this paper.

According to the generated asymmetrical cloud, a mutation certainty degree *μ*_*m*_ is used to decide the mutated result of antibody. In the AC-based mutation operator, the searching step length adjustment mechanism can improve the solution accuracy, which is determined by the numerical characteristics of the asymmetrical cloud. The pseudocode of the AC-based mutation operator is shown in Pseudocode 2 and its corresponding asymmetrical cloud is depicted in Figure 3. After the mutation process, the antibody with the highest affinity is selected to enter the next generation.

### 3.3. Suppression Operator

**Figure 4:**
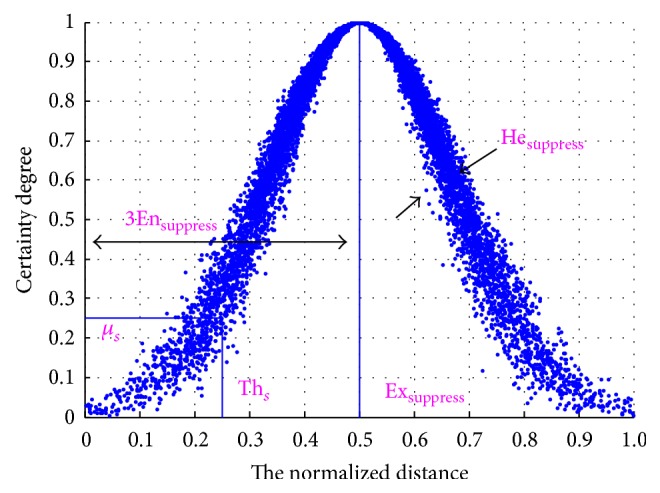
The normal similarity cloud-based suppression operator (cloud chart) (see also Pseudocode 3).

Once the average affinity at the current generation is not significantly different from the previous one, a* normal similarity cloud-based suppression operator (NSC-based suppression operator)* will be activated. In the suppression phase, a normal similarity cloud shown in Figure 4 and Pseudocode 3 is determined by the Euclidean distance *D*(*i*, *j*) between the *i*th and *j*th antibodies. According to the backward cloud generator [16], the numerical characteristics of the normal similarity cloud can be expressed as

(7) Exsuppress=∑i=1N∑j=1NDi,jCN2,Ensuppress=π2×∑i=1N∑j=1NDi,j−ExsuppressCN2,Hesuppress=∑i=1N∑j=1NDi,j−ExsuppressCN2−1.

In this normal similarity cloud, Ex_suppress_ is the sample average which lies in the center of the cloud, En_suppress_ is derived from Ex_suppress_ and denotes the range of the *D*(*i*, *j*), and He_suppress_ represents the dispersion of *D*(*i*, *j*). According to the generated normal similarity cloud, a suppression certainty degree *μ*_*s*_ is used to determine the threshold Th_*s*_. The pseudocode of NSC-based suppression operator is shown in Pseudocode 3 and its corresponding normal similarity cloud in suppression operator is illustrated in Figure 4.

For any couple of antibodies whose Euclidean distance is less than a threshold Th_*s*_, the worse one in affinity will be suppressed or removed. After the suppression process, the number of the antibodies will decrease significantly. Thus, a number of randomly generated antibodies are recruited to keep the scale and the diversity of antibodies. Repeat the above iterative process until the termination condition is satisfied.

### 3.4. The Algorithmic Flowchart

**Figure 5:**
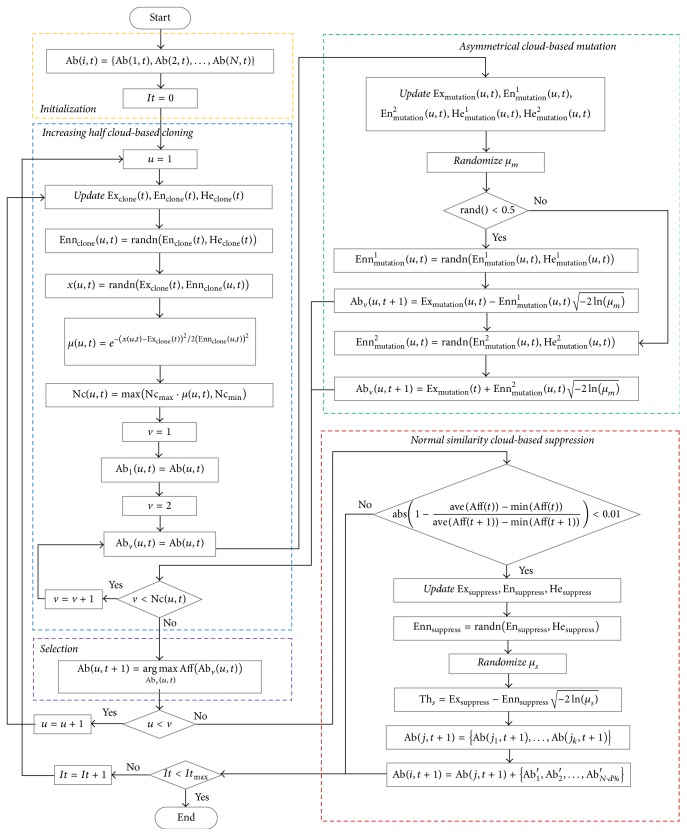
Flowchart of the proposed AINet-CM algorithm.

The flowchart of the proposed AINet-CM algorithm is shown in Figure 5. Observed from Figure 5, the technical procedure of AINet-CM includes the following:

1. Initializing the antibody population and parameter setting.
2. Executing the increasing half cloud-based cloning operator.
3. Executing the asymmetrical cloud-based mutation operator.
4. Selecting the candidate antibodies with the highest affinity.
5. Executing the normal similarity cloud-based suppression operator.
6. While the stop criterion is met, the procedure is finished; otherwise turn to Step (2).

## 4. Numerical Simulations and Results

In this section, a series of numerical simulations are executed to examine the performance of the proposed AINet-CM algorithm. Comparisons are made among the proposed AINet-CM algorithms, opt-aiNet, IA-AIS, and AAIS-2S in solution accuracy and the convergence speed.

### 4.1. Benchmark Functions

For the sake of numerical evaluations, five benchmark functions are selected from* CEC2005* [23] and listed as follows.

1. *F*_1_ Shifted Sphere Function: unimodal
2. *F*_2_ Shifted Schwefel's Problem 1.2: unimodal
3. *F*_6_ Shifted Rosenbrock's Function: multimodal
4. *F*_9_ Shifted Rastrigin's Function: multimodal
5. *F*_12_ Schwefel's Problem 2.13: multimodal

### 4.2. Parameter Settings

In the numerical simulations, the initialized population size of the four algorithms is set to 100, the maximum generation is 1000 for 2D or 5000 for 10D and 30D, and all the simulations are repeated for 50 trials. For the sake of clarity, all the parameters of four algorithms are listed in Table 1.

### 4.3. Simulation Results and Analyses

#### 4.3.1. Performance Analyses in Solution Accuracy

The simulation results optimized by four algorithms in 2D, 10D, and 30D are presented in Tables 2–4, respectively. The resulting error indices derived from 50 replications consist of the best, the worst, the average, and the standard deviation (std) of the error. The best results among these algorithms are marked in bold.

For 2D optimization, as shown in Table 2, the proposed AINet-CM algorithm can capture the global optimum of each benchmark function except for *F*_6_ in every trial. For instance, in optimizing functions *F*_1_, *F*_2_, *F*_9_, and *F*_12_, AINet-CM is always capable of finding their desired optima for every replication. As a result, the four statistical error indices are all equal to be 0. On the other hand, the other three algorithms except AAIS-2S almost do not reach the desired optima of these functions. The only exception is that AAIS-2S can get the desired optimum of *F*_1_. For another instance, in optimizing function *F*_6_, AINet-CM obtains the worst error 4.971701*e* − 004, which is greater than 6.500257*e* − 006 by IA-AIS, but the average and std of the error obtained by AINet-CM are much smaller.

For 10D optimization, as shown in Table 3, the proposed AINet-CM algorithm almost performs the best in all four statistical indices among four algorithms, whatever the optimized function is. For example, in optimizing functions *F*_1_, *F*_6_, *F*_9_, and *F*_12_, AINet-CM is always able to reach less index value in error than the other three algorithms. Specifically speaking, in optimizing *F*_1_ and *F*_9_, AINet-CM actually reaches their desired optima, that is, the best of error 0. For another example, in optimization function *F*_12_, AINet-CM gets 1.271222*e* − 001 and IA-AIS does 6.819255*e* − 002 in terms of the worst error. Seen from Table 3, it is clear that AINet-CM loses to IA-AIS only in the worst error, but AINet-CM defeats IA-AIS in the other error indices.

For 30D optimization, as shown in Table 4, AINet-CM outperforms other algorithms except for *F*_12_. To be specific, in optimizing *F*_1_, *F*_2_, *F*_6_, and *F*_9_, AINet-CM obtains the smallest index value in the best, worst, average, and std of error among four compared algorithms. Specially in optimizing *F*_1_, AINet-CM gets relatively high-accuracy solution because it has the best error of 5.684342*e* − 012, the worst error of 5.684342*e* − 008, the average error of 5.684342*e* − 009, and the std of error of 3.54465*e* − 010. As an exception, in optimizing *F*_12_, AINet-CM performs worse in the worst error, the average error, and the std error than opt-aiNet, but the best error captured by AINet-CM is better than that by other algorithms.

Seen from these results, it is obvious that the searching step length adjustment mechanism increases the solution accuracy of AIS. The improvements in the average error and the std of error prove that this mechanism is able to efficiently and dynamically adjust the searching range in order to guarantee the antibodies' evolution in affinity even when the error is rather small. However, according to the worst error in optimizing 10D function *F*_2_ and the results in 30D function *F*_12_, it is similar to other algorithms that AINet-CM still has small potential risk in getting trapped into some local optima.

#### 4.3.2. Performance Analyses in Convergence Speed

**Figure 6:**
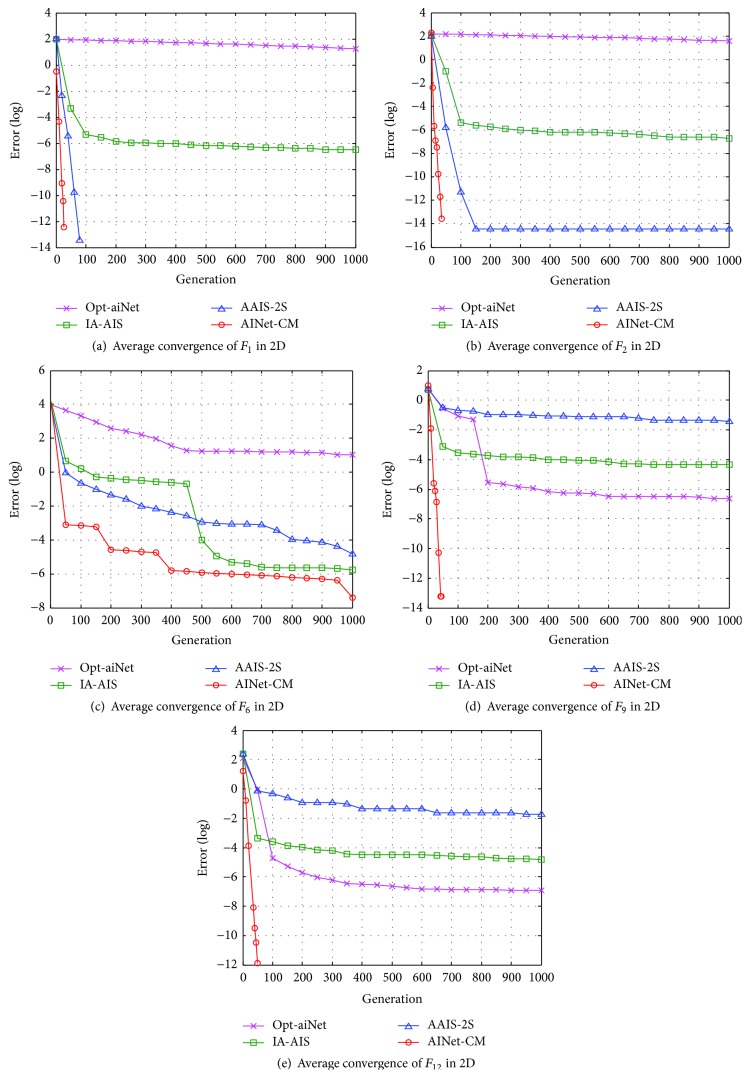
Average convergence processes of five 2D benchmark functions.

**Figure 7:**
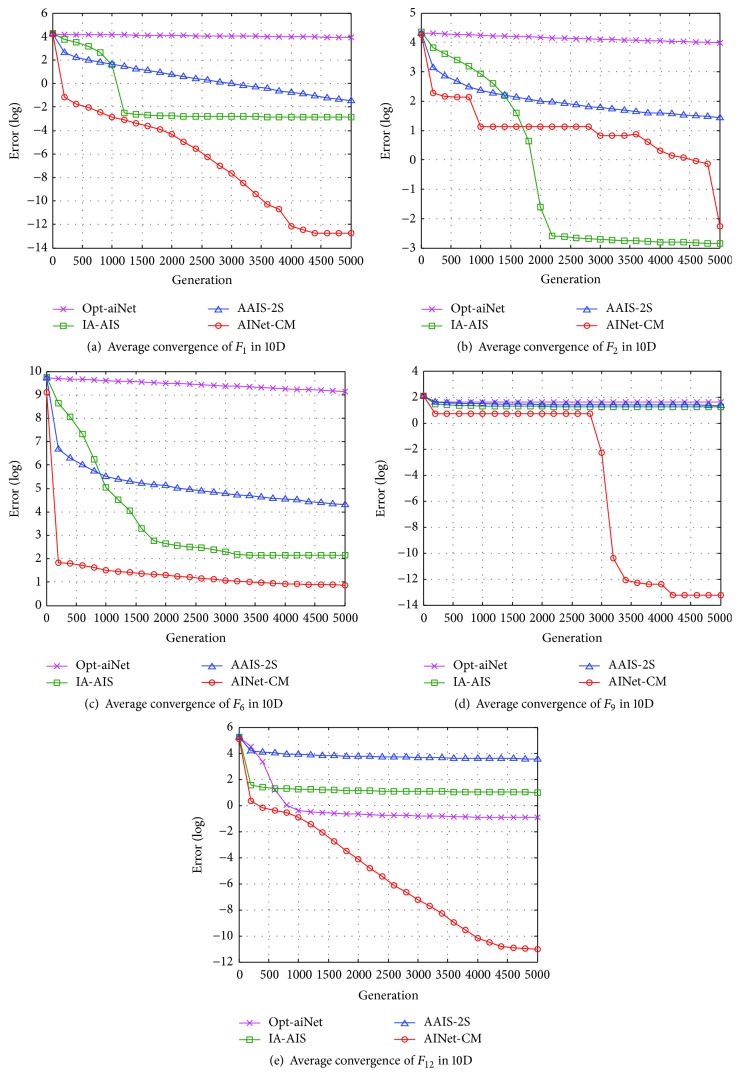
Average convergence processes of five 10D benchmark functions.

**Figure 8:**
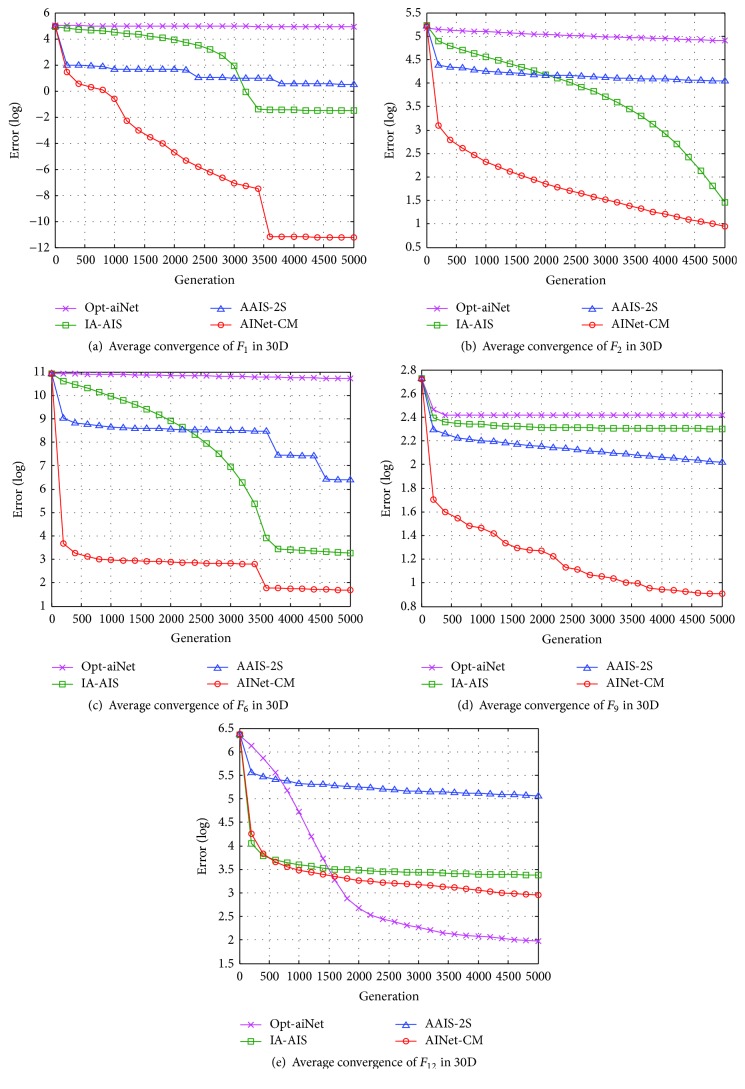
Average convergence processes of five 30D benchmark functions.

Figures 6–8 present the average convergence processes for the five benchmark functions. It is clear that the proposed AINet-CM algorithm has much faster convergence speed than the other three algorithms in optimizing 2D, 10D, and 30D benchmark functions in most situations.

For 2D optimization, as shown in Figures 6(a)–6(e), the proposed AINet-CM algorithm can obtain the optima easily within only 100 generations in optimizing functions *F*_1_, *F*_2_, *F*_9_, and *F*_12_. For function *F*_6_, although the AINet-CM algorithm cannot get the optima within 1000 generations, it still has much faster convergence speed than the other algorithms. For 10D optimization, as shown in Figures 7(a)–7(e), it is obvious that the proposed AINet-CM algorithm outperforms the other three algorithms in optimizing functions *F*_1_, *F*_6_, *F*_9_, and *F*_12_. Especially for function *F*_2_, AINet-CM is a little slower convergence speed than IA-AIS and AAIS-2S in the earlier phase. However, after 2200 generations, AINet-CM still keeps faster speed than the other three algorithms. This is because that a tradeoff is made between the solution accuracy and the convergence speed. For 30D optimization, as shown in Figures 8(a)–8(e), the proposed AINet-CM algorithm has better convergence speed in optimizing functions *F*_1_, *F*_2_, *F*_6_, and *F*_9_. Especially for function *F*_12_, seen from Figure 8(e), AINet-CM can get the best optima, although AINet-CM has a slower average convergence speed than opt-aiNet.

Observed from the convergence curves in Figures 6–8, the convergence speed of AINet-CM is improved significantly due to usage of cloud model measure. The results indicate that the diversity and stability of cloud model increase the probability of antibodies to evolve towards the global optimum and further decrease the risk in falling into the local optima.

## 5. Applications in FIR Filter Design and PID Parameter Tuning

### 5.1. Application in Designing FIR Filter

Finite impulse response (FIR) filter is known as a nonrecursive filter that the response due to an impulse input will decay within finite time [24]. Due to the lack of feedback and the symmetrical characteristics about the center tap position, FIR filter can be guaranteed to have strict linear phase at all frequencies. In addition, FIR filter has many desirable characteristics such as stability, robustness, and digital implementation. Therefore, FIR filter has broad applications in communications, image processing, pattern recognition, and so forth.

The *z* transform of an *Q*-point FIR filter is characterized by

(8) $$\begin{array}{c} H\left(\;z\;\right)=\overset{Q-1}{\underset{q=0}{\sum }}h\left(\;q\;\right)z^{-q}, \end{array}$$

where *h*(*q*) is the impulse response and is finite, that is, 0 ≤ *q* ≤ *Q* − 1. So the frequency response of the FIR filter can be calculated as

(9) $$\begin{array}{c} H\left(\;e^{j\omega_{k}}\;\right)=\overset{M-1}{\underset{q=0}{\sum }}h\left(\;q\;\right)e^{-j\omega_{k}q}, \end{array}$$

where *ω*_*k*_ = 2*πk*/*M* and *H*(*e*^*jω*_*k*_^) is the Fourier transform complex vector and the frequency is sampled in [0, *π*] with *M* points. Hence, the optimal filter design method is employed to minimize a particular error. The least squared error can be obtained by

(10) E∑k=1MHidealejωk−Hdesignedejωk2=∑k=1M∑q=0Q−1hqejωkq−Hdesignedejωk2,

where *H*_ideal_(*e*^*jω*_*c*_^) represents the magnitude response of the ideal filter and *H*_designed_(*e*^*jω*_*c*_^) represents the filter to be designed. To design a FIR filter is focused on determining the set of {*h*(0), *h*(1),…, *h*(*Q* − 1)} to minimize the least squared error.

In this application, an ideal band-pass FIR filter is to be designed in which the frequency response is expected as

(11) Hidealejω=1,0.32π≤ω≤0.68π,0,0≤ω<0.32π  or  0.68π<ω≤π.

Moreover, *M* is set to be 100. The proposed AINet-CM algorithm is compared with opt-aiNet, IA-AIS, and AAIS-2S in searching the least square error, and the maximum of generation is 1000.

The simulation results reached by four algorithms with *Q* = 10 and *Q* = 30 are presented in Tables 5-6, respectively. The results include the best, worst, average, and standard deviation (std) of the error, and the best results are typed in bold. Seen from Tables 5-6, the results obtained by AINet-CM are smaller than the other three algorithms. To be specific, with *Q* = 10, AINet-CM harvests the best error of 3.436117, the worst error of 3.438717, the average error of 3.436537, and the std of error of 7.202963*e* − 004. With *Q* = 30, AINet-CM reaches the best error of 1.122552, the worst error of 1.122616, the average error of 1.122571, and the std of error of 1.661895*e* − 005. In particular, whatever *Q* equals 10 or 30, the std of error obtained by AINet-CM is always the smallest, which means the most consistent solution among replications. It is clear that AINet-CM is capable of capturing better solutions than opt-aiNet, IA-AIS, and AAIS-2S.

**Figure 9:**
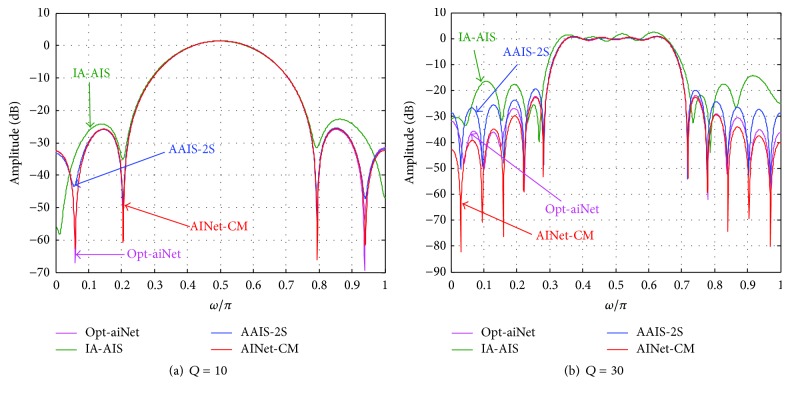
The frequency response of the band-pass filter.

Figure 9 illustrates the frequency responses by using the best solutions in Tables 5-6. Seen from Figure 9(a) when *Q* equals 10, the amplitude responses produced by four algorithms are similar in the pass band, while the frequency responses produced by opt-aiNet and AINet-CM outperform the other algorithms in the stop band because there exist smaller amplitudes in the figure. Observed from Figure 9(b) when *Q* equals 30, all the algorithms except IA-AIS produce similar frequency responses to the ideal one in the pass band. However, AINet-CM produces much smaller amplitude response (in red) than the other three algorithms in the stop band. Hence, it would be concluded that the band-pass filter designed by AINet-CM is superior to the other three algorithms.

### 5.2. Application in Tuning PID Controller Parameters

**Figure 10:**
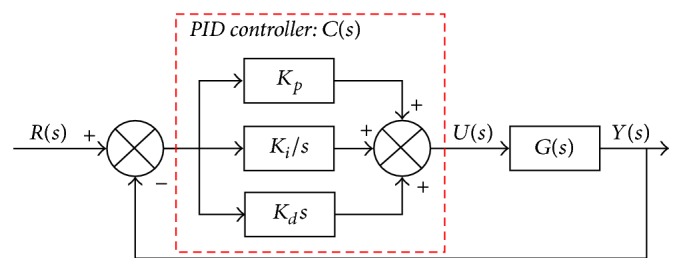
The block diagram of the PID control system.

The proportional-integral-derivative (PID) controllers are the most popular controllers in the process industries [25]. Although PID controllers are characteristic of effectiveness, strong robustness, and simple implementation, they are poorly tuned. Therefore, parameter tuning *s* is crucial to PID controller design. The closed-loop diagram of the PID control system is illustrated in Figure 10.

In Figure 10, *R*(*s*), *E*(*s*), *U*(*s*), and *Y*(*s*) are the reference input, the error input, the controller output, and the system output (also the feedback variable), respectively, *C*(*s*) is the transfer function of PID controller and *G*(*s*) is the controlled object transfer function, and *K*_*p*_, *K*_*i*_, and *K*_*d*_ are the proportional, integral, and derivative parameters of the PID controller, respectively. So the transfer function *C*(*s*) is expressed as

(12) $$\begin{array}{c} C\left(\;s\;\right)=K_{p}+K_{i}\frac{1}{s}+K_{d}s. \end{array}$$

In this application, the object transfer function *G*(*s*) is modeled as

(13) $$\begin{array}{c} G\left(\;s\;\right)=\frac{1}{s^{4}+6s^{3}+11s^{2}+6s}. \end{array}$$

Activated by a step pulse stimulus, the parameters *K*_*p*_, *K*_*i*_, and *K*_*d*_ of PID controller are required to be tuned to meet the desired performance criteria of the entire control system. In this paper, two indices, the integral of absolute magnitude of the error (IAE) and the integral of time-weight absolute error (ITAE), are selected as the performance criteria. They are mathematically expressed by

(14) IAE=∫0∞etdt,ITAE=∫0∞tetdt,

where *e*(*t*) is the negative feedback control system error. In addition, the size of initialized population is 80 and the maximum number of generation is 100 in the simulations.

Tables 7-8 present the simulation results optimized by the four algorithms. Seen from these tables, the IAE value and the ITAE value optimized by AINet-CM are 1.3204049*e* + 002 and 1.5664397*e* + 001, respectively, which are smaller than those by the other three algorithms. It means that AINet-CM obtains better PID parameters than the other algorithms.

**Figure 11:**
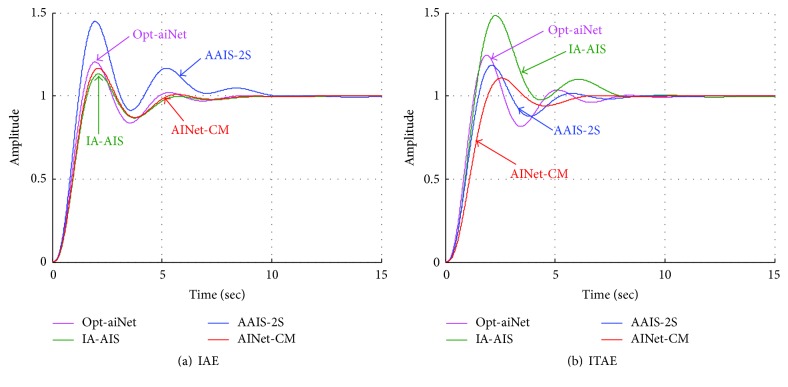
Step response of PID controller.

For the sake of clear observations, Figure 11 shows the step response curves corresponding to four algorithms, which are marked in different colors such as pink (opt-aiNet), green (IA-AIS), blue (AAIS-2S) and red (AINet-CM). Compared to the other three algorithms, seen from Figure 11, the proposed AINet-CM algorithm has smaller overshoot or less settle time in both IAE and ITAE measures. Specifically speaking, PID controller obtains a better ideal step response curve which is tuned by AINet-CM when ITAE is used. To be specific, in Figure 11(a), AINet-CM harvests the least settle time of 5.5 seconds even if its overshoot and rise time are not the smallest. In Figure 11(b), the proposed AINet-CM algorithm obtains the overshoot of 10% and the settle time of 6 seconds while the other algorithms get larger overshoot and settle time. These results indicate that the proposed AINet-CM algorithm is more capable of finding the optimal parameters in PID controller tuning.

## 6. Conclusion

In this paper, an artificial immune network based on cloud model (AINet-CM) is proposed for complex optimization problems. By introducing the cloud model, the proposed AINet-CM algorithm is formed by redesigning three immune operators, that is, the increasing half cloud-based cloning operator, the asymmetrical cloud-based mutation operator, and the normal similarity cloud-based suppression operator. A series of numerical simulations are executed, and the resulting data indicate that the proposed AINet-CM algorithm has much less error in solution accuracy and much faster convergence speed in most situations by comparison with opt-aiNet, IA-AIS, and AAIS-2S in optimizing 2D, 10D, and 30D functions. Further, the simulation results in FIR filter design show that the proposed AINet-CM algorithm provides an efficient and superior approach for digital filter design. The simulation results in tuning PID parameters prove that PID controller optimized by AINet-CM is superior to those by opt-aiNet, IA-AIS, and AAIS-2S.

## Figures and Tables

**Pseudocode 1:**
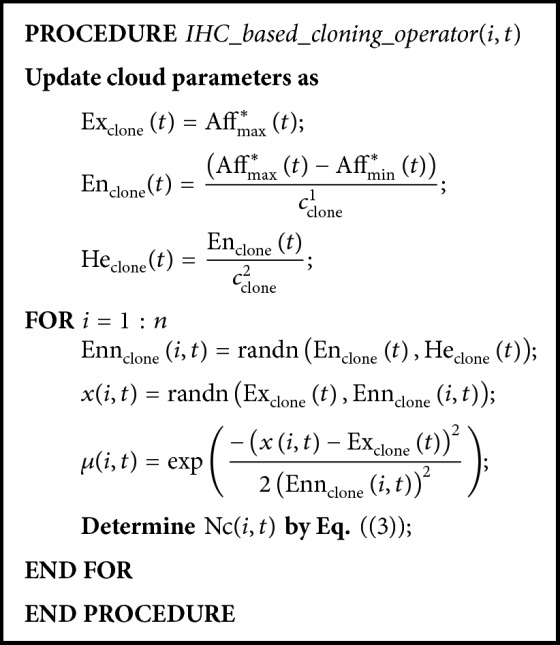


**Pseudocode 2:**
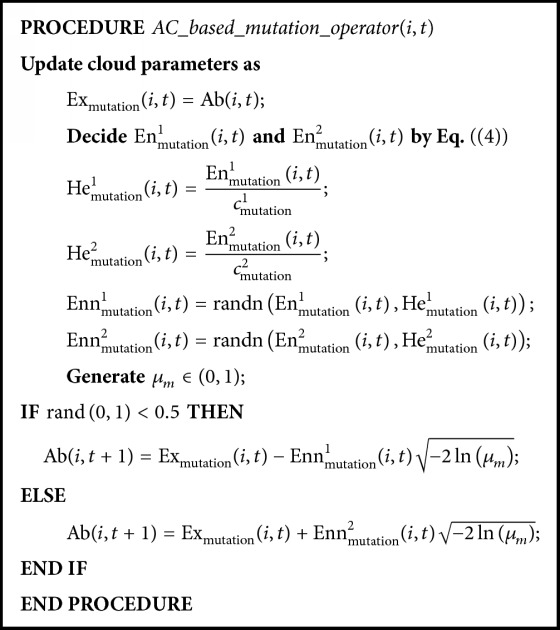


**Pseudocode 3:**
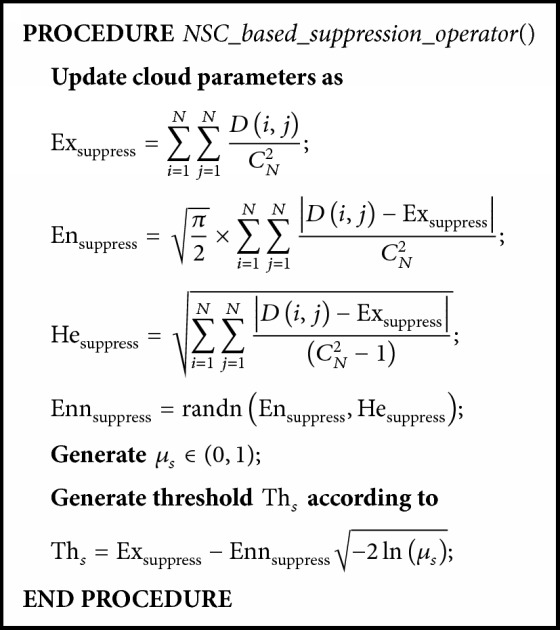


**Table 1:** Parameter settings of four algorithms.

Algorithm	Cloning operator	Mutation operator	Suppression operator	Others
Opt-aiNet	Nc = 20	*β* = 100	*σ*_*s*_ = 0.2 *d* = 40%	—

IA-AIS	Nc_max_ = 20 Nc_min_ = 4 *n* = 2	*γ* = 2 *η* = 0.33	*ξ* = 0.1	—

AAIS-2S	Nc_max_ = 20 Nc_min_ = 4 *n* = 2	*η* = 0.33	*ξ* = 0.1	*p*_*e*_ = 0.25 *N*_update_ = 10

AINet-CM	Nc_max_ = 20 Nc_min_ = 4 *c*_clone_^1^ = 3 *c*_clone_^2^ = 10	*K* = 0.5 *β*_min_ = 10*e* − 016 *c*_mutation_^1^ = 10 *c*_mutation_^2^ = 10 *β*_0_ = 0.3	*μ*_*s*_ = 0.3	—

**Table 2:** The optimization simulation results in 2D.

Benchmark	Algorithm	Best	Worst	Average	Std
*F*_1_	Opt-aiNet	4.075673*e* − 010	2.322654*e* + 002	2.134278*e* + 001	3.815297*e* + 001
	IA-AIS	1.142894*e* − 009	1.272159*e* − 006	2.081269*e* − 007	2.554790*e* − 007
	AAIS-2S	0	0	0	0
	AINet-CM	0	0	0	0

*F*_2_	Opt-aiNet	8.334382*e* − 010	7.423637*e* + 002	4.424344*e* + 001	1.172033*e* + 002
	IA-AIS	5.649667*e* − 010	6.458520*e* − 007	2.181431*e* − 007	1.829096*e* − 007
	AAIS-2S	0	5.684342*e* − 014	1.136868*e* − 015	8.038873*e* − 015
	AINet-CM	0	0	0	0

*F*_6_	Opt-aiNet	1.601273*e* − 008	6.982493*e* + 001	1.332500*e* + 001	1.453700*e* + 001
	IA-AIS	7.696849*e* − 008	6.500257**e** − 006	1.330222*e* − 006	1.461563*e* − 006
	AAIS-2S	0	2.765851*e* − 002	5.749097*e* − 004	3.910412*e* − 003
	AINet-CM	0	4.971701*e* − 004	7.306988**e** − 007	1.300343**e** − 006

*F*_9_	Opt-aiNet	6.197070*e* − 010	1.484871*e* − 006	3.183155*e* − 007	3.302604*e* − 007
	IA-AIS	2.181800*e* − 007	9.205743*e* − 005	2.840228*e* − 005	2.386444*e* − 005
	AAIS-2S	0	5.169959*e* − 001	5.521124*e* − 002	1.051350*e* − 001
	AINet-CM	0	0	0	0

*F*_12_	Opt-aiNet	7.541985*e* − 010	5.799400*e* − 007	6.917494*e* − 008	9.323257*e* − 008
	IA-AIS	8.398080*e* − 007	1.160427*e* − 004	2.146887*e* − 005	2.347865*e* − 005
	AAIS-2S	0	3.548364*e* − 001	2.313225*e* − 002	6.041159*e* − 002
	AINet-CM	0	0	0	0

**Table 3:** The optimization simulation results in 10D.

Benchmark	Algorithm	Best	Worst	Average	Std
*F*_1_	Opt-aiNet	2.677496*e* + 003	1.447263*e* + 004	8.249127*e* + 003	3.058784*e* + 003
	IA-AIS	6.222499*e* − 004	2.049487*e* − 003	1.297094*e* − 003	3.459514*e* − 004
	AAIS-2S	6.107627*e* − 004	3.282865*e* − 001	3.606023*e* − 002	5.323906*e* − 002
	AINet-CM	0	1.608669**e** − 011	9.518249**e** − 013	2.964222**e** − 012

*F*_2_	Opt-aiNet	2.332892*e* + 003	1.695573*e* + 004	9.691981*e* + 003	3.404834*e* + 003
	IA-AIS	6.819255*e* − 004	6.819255**e** − 002	1.439519*e* − 003	3.506100*e* − 004
	AAIS-2S	5.654689*e* + 000	7.858485*e* + 001	2.721496*e* + 001	1.389431*e* + 001
	AINet-CM	5.488060**e** − 004	1.271222*e* − 001	1.024232**e** − 003	3.271727**e** − 004

*F*_6_	Opt-aiNet	4.059666*e* + 007	4.284734*e* + 009	1.364979*e* + 009	9.506303*e* + 008
	IA-AIS	1.919524*e* + 000	1.112680*e* + 003	1.404718*e* + 002	2.618577*e* + 002
	AAIS-2S	3.556545*e* + 003	9.837639*e* + 004	1.989282*e* + 004	1.866355*e* + 004
	AINet-CM	2.994048**e** − 001	5.290081**e** + 001	1.066962**e** + 000	1.187079**e** + 001

*F*_9_	Opt-aiNet	1.592055*e* + 001	6.268373*e* + 001	3.993963*e* + 001	1.043980*e* + 001
	IA-AIS	7.114565*e* + 000	2.330663*e* + 001	1.634941*e* + 001	3.830072*e* + 000
	AAIS-2S	1.555034*e* + 001	2.822564*e* + 001	2.307381*e* + 001	2.660738*e* + 000
	AINet-CM	0	1.215067**e** − 006	2.494085**e** − 008	1.718036**e** − 007

*F*_12_	Opt-aiNet	4.483347*e* − 002	1.999181*e* − 001	1.139036*e* − 001	3.514291*e* − 002
	IA-AIS	6.018482*e* + 000	1.337908*e* + 001	9.997727*e* + 000	1.814684*e* + 000
	AAIS-2S	9.189471*e* + 002	7.018537*e* + 003	3.657929*e* + 003	1.363907*e* + 003
	AINet-CM	4.206413**e** − 012	2.696698**e** − 003	3.464392**e** − 004	7.601388**e** − 004

**Table 4:** The optimization simulation results in 30D.

Benchmark	Algorithm	Best	Worst	Average	Std
*F*_1_	Opt-aiNet	7.364028*e* + 004	9.043620*e* + 004	8.163091*e* + 004	6.326750*e* + 003
	IA-AIS	2.845131*e* − 002	3.355290*e* − 002	3.033896*e* − 002	1.764107*e* − 003
	AAIS-2S	3.594068*e* − 001	6.647420*e* + 003	5.031004*e* + 001	1.048068*e* + 002
	AINet-CM	5.684342**e** − 012	5.684342**e** − 008	5.684342**e** − 009	3.54465**e** − 010

*F*_2_	Opt-aiNet	4.421657*e* + 004	1.179479*e* + 005	8.084211*e* + 004	2.078686*e* + 004
	IA-AIS	1.351844*e* − 001	3.844238*e* + 000	2.888746*e* + 001	1.203280*e* + 002
	AAIS-2S	8.063256*e* + 003	1.462887*e* + 004	1.114811*e* + 004	1.691137*e* + 003
	AINet-CM	3.001361**e** + 000	2.047495**e** + 001	8.902851**e** + 000	3.844238**e** + 000

*F*_6_	Opt-aiNet	2.167594*e* + 010	8.408124*e* + 010	5.198204*e* + 010	1.449699*e* + 010
	IA-AIS	2.945045*e* + 001	1.243707*e* + 004	1.887542*e* + 003	3.299679*e* + 003
	AAIS-2S	1.759388*e* + 005	3.462921*e* + 008	2.484637*e* + 006	5.201979*e* + 007
	AINet-CM	2.198607**e** + 000	2.419968**e** + 002	4.786265**e** + 001	6.821265**e** + 001

*F*_9_	Opt-aiNet	2.356387*e* + 002	2.895655*e* + 002	2.608678*e* + 002	1.794656*e* + 001
	IA-AIS	1.427379*e* + 002	2.238784*e* + 002	2.004291*e* + 002	2.165710*e* + 001
	AAIS-2S	8.164155*e* + 001	1.216505*e* + 002	1.042647*e* + 002	1.211470*e* + 001
	AINet-CM	3.979836**e** + 000	2.089416**e** + 001	8.046239**e** + 000	5.393961**e** + 000

*F*_12_	Opt-aiNet	3.661858*e* + 001	1.333772**e** + 002	8.587916**e** + 001	3.176119**e** + 001
	IA-AIS	1.935926*e* + 003	2.898353*e* + 003	2.290360*e* + 003	3.430398*e* + 002
	AAIS-2S	8.728301*e* + 004	1.370337*e* + 005	1.149788*e* + 005	1.681238*e* + 004
	AINet-CM	5.106115**e** + 000	2.580782*e* + 003	9.023991*e* + 002	7.573875*e* + 002

**Table 5:** Simulation results in FIR filter designing (*Q* = 10).

Algorithm	Best	Worst	Average	Std
Opt-aiNet	3.436566*e* + 000	3.439407*e* + 000	3.436971*e* + 000	2.458096*e* − 003
IA-AIS	3.460142*e* + 000	3.542927*e* + 000	3.510426*e* + 000	2.657726*e* − 002
AAIS-2S	3.436307*e* + 000	3.471676*e* + 000	3.447269*e* + 000	9.706593*e* − 003
AINet-CM	3.436117**e** + 000	3.438717**e** + 000	3.436537**e** + 000	7.202963**e** − 004

**Table 6:** Simulation results in FIR filter designing (*Q* = 30).

Algorithm	Best	Worst	Average	Std
Opt-aiNet	1.131956*e* + 000	1.137890*e* + 000	1.135113*e* + 000	1.818352*e* − 003
IA-AIS	2.716332*e* + 000	3.592075*e* + 000	3.112379*e* + 000	2.250955*e* − 001
AAIS-2S	1.132001*e* + 000	1.134296*e* + 000	1.133744*e* + 000	1.525802*e* − 003
AINet-CM	1.122552**e** + 000	1.122616**e** + 000	1.122571**e** + 000	1.661895**e** − 005

**Table 7:** Simulation results in PID parameter tuning (IAE).

Algorithm	PID parameters			IAE
	*K*_*p*_	*K*_*i*_	*K*_*d*_	
Opt-aiNet	1.4629331*e* + 001	6.4300879*e* + 000	5.1237192*e* − 003	1.3618251*e* + 002
IA-AIS	1.2500524*e* + 001	5.4443793*e* + 000	2.6231018*e* − 003	1.3625645*e* + 002
AAIS-2S	1.6155824*e* + 001	1.0210501*e* + 001	2.6938200*e* + 000	1.7934799*e* + 002
AINet-CM	1.1785053**e** + 001	5.9124436**e** + 000	6.2479890**e** − 004	1.3204049**e** + 002

**Table 8:** Simulation results in PID parameter tuning (ITAE).

Algorithm	PID parameters			ITAE
	*K*_*p*_	*K*_*i*_	*K*_*d*_	
Opt-aiNet	1.5915666*e* + 001	7.0229236*e* + 000	2.9382817*e* − 003	2.2933311*e* + 001
IA-AIS	1.2001105*e* + 001	1.0006757*e* + 001	2.7380186*e* + 000	3.8046764*e* + 001
AAIS-2S	1.2471515*e* + 001	6.4891158*e* + 000	9.2537522*e* − 005	1.8178800*e* + 001
AINet-CM	8.7795676**e** + 000	5.4957820**e** + 000	1.1497691**e** − 004	1.5664397**e** + 001
